# Engineering *Yarrowia lipolytica* to Produce Itaconic Acid From Waste Cooking Oil

**DOI:** 10.3389/fbioe.2022.888869

**Published:** 2022-04-25

**Authors:** Lanxin Rong, Lin Miao, Shuhui Wang, Yaping Wang, Shiqi Liu, Zhihui Lu, Baixiang Zhao, Cuiying Zhang, Dongguang Xiao, Krithi Pushpanathan, Adison Wong, Aiqun Yu

**Affiliations:** ^1^ State Key Laboratory of Food Nutrition and Safety, Key Laboratory of Industrial Fermentation Microbiology of the Ministry of Education, Tianjin Key Laboratory of Industrial Microbiology, College of Biotechnology, Tianjin University of Science and Technology, Tianjin, China; ^2^ Food, Chemical and Biotechnology Cluster, Singapore Institute of Technology, Dover, Singapore

**Keywords:** itaconic acid, *Y. lipolytica*, waste cooking oil, peroxisome, subcellular engineering

## Abstract

Itaconic acid (IA) is a high-value organic acid with a plethora of industrial applications. In this study, we seek to develop a microbial cell factory that could utilize waste cooking oil (WCO) as raw material for circular and cost-effective production of the abovementioned biochemical. Specifically, we expressed cis-aconitic acid decarboxylase (CAD) gene from *Aspergillus terreus* in either the cytosol or peroxisome of *Yarrowia lipolytica* and assayed for production of IA on WCO. To further improve production yield, the 10 genes involved in the production pathway of acetyl-CoA, an intermediate metabolite necessary for the synthesis of cis-aconitic acid, were individually overexpressed and investigated for their impact on IA production. To minimize off-target flux channeling, we had also knocked out genes related to competing pathways in the peroxisome. Impressively, IA titer up to 54.55 g/L was achieved in our engineered *Y. lipolytica* in a 5 L bioreactor using WCO as the sole carbon source.

## Introduction

Carboxylic acids are important building blocks in the chemical industry. Among them, itaconic acid (IA) is favorably listed by the US Department of Energy as one of top 12 biochemical to be produced from renewable resources (Werpy and Petersen, 2004), with a forecasted market potential of $260 million in 2025 (Sriariyanun, 2019). IA is an unsaturated dicarboxylic acid that is characteristically stable in acidic, neutral and moderately alkaline conditions. Due to its advantageous properties, IA is often used as a co-monomer in the manufacture of synthetic fibers, coatings, adhesives, thickeners and binders (Willke and Vorlop, 2001; Zhao et al., 2018), and as substitutes for petrochemical-based acrylic or methacrylic acids (Nuss and Gardner, 2013). Traditionally, to meet the growing demand for IA, industries resort to fossil resources through petrochemical refinery processes to produce IA at scale. However, these methods often suffer from low efficiency and generate large amount of waste in the process, such as spent heavy metal catalysts and organic solvents (Krull et al., 2017). Furthermore, fossil resources are finite and will eventually be depleted. For these reasons, bio-based production of IA using microbial cell factories are increasingly being pursued.

Filamentous fungi such as *Aspergillus terreus* (Kuenz et al., 2012), *Ustilago maydis* (Geiser et al., 2016) and *Ustilago cynodontis* (Hosseinpour Tehrani et al., 2019b) have been demonstrated to naturally produce IA at high titers. In one example, the fermentation of *A. terreus* at industrial scale is able to generate a titer of 160 g/L IA (Krull et al., 2017), a value that is close to the theoretical yield. In another example, up to 220 g/L IA was achieved by fermentation of *U. maydis* (Hosseinpour Tehrani et al., 2019a). Despite having high production titers, current bioprocesses involving filamentous fungi are not without challenges. Critically, the highly branched mycelial filaments of filamentous fungi give rise to high broth viscosity during fermentation, leading to poor aeration and mixing in stirred-tank bioreactors (Kubicek et al., 2011; Porro and Branduardi, 2017). Increasing impeller speed, on the other hand, is not an option due to the shear-sensitive nature of filamentous fungi. Moreover, fermentation of most filamentous fungi requires the addition of alkali to maintain a neutral pH condition which is a cause of concern as this increases the probability of bacterial contamination during cultivation (Cui et al., 2017; Li et al., 2021). To circumvent issues associated with filamentous fungi bioprocessing, scientists have applied systems metabolic engineering principles to enable heterologous production of IA in several strains of bacteria and yeasts (Table 1).

**TABLE 1:** Representative examples of IA production in engineered microbial hosts.

Parental strain	Engineering strategy	Fermentation condition	Carbon source	Titer	References
*E. coli*	*CAD*↑*, CS*↑*, ICD*↓, *ICL*Δ*, PTA*Δ, *PYK*Δ*, SUCS*Δ	Fed-batch bioreactor	Glucose and glutamic acid	32.00 g/L	Harder et al. (2016)
	*CAD*↑*, ACO*↑*, ICD*Δ	Fed-batch bioreactor	LB + Glucose	4.34 g/L	Okamoto et al. (2014)
	*CAD*↑*, CS*↑*, ACO*↑*, PTA*Δ*, LDH*Δ	Bioreactor	LB + Glucose	0.69 g/L	Vuoristo et al. (2015)
*S. cerevisiae*	*CAD*↑*, ADE3*Δ, *BNA2*Δ, *TES1*Δ	Large-scale bioreactor	Glucose	0.17 g/L	Blazeck et al. (2014)
*Halomonas bluephagenesis*	*CAD*↑*, ACO*↑*, ICD*↓	Batch shake flask	Citrate	63.60 g/L	Zhang et al. (2021a)
*Corynebacterium glutamicum*	*CAD*↑*, MALE*↑*, ICD*↓	Shake flask	Glucose	7.80 g/L	Otten et al. (2015)
*Pichia kudriavzevii*	*CAD*↑*, MTT*↑*, ICD*Δ	Fed-batch bioreactor	Glucose	1.23 g/L	Sun et al. (2020)
*Y. lipolytica*	*CAD*↑*, ACO*↑, *AMPD*↓	Bioreactor	Glucose	4.60 g/L	Blazeck et al. (2015)
	*CAD*↑*, MTT*↑	Fed-batch bioreactor	Glucose	22.02 g/L	Zhao et al. (2019)
	*CAD-ePTS1*↑*, POT1*↑*, ICL*Δ	Bioreactor	Waste cooking oil	54.55 g/L	This study

↑, Gene overexpression; ↓, gene knockdown; Δ, gene knockout; *CAD*, cis-aconitic acid decarboxylase gene; *CS*, citrate synthase gene; *ICD*, isocitrate dehydrogenase gene; *ICL*, isocitrate lyase gene; *PTA*, phosphate acetyltransferase gene; *PYK*, pyruvate kinase gene; *SUCS*, succinyl-CoA synthetase gene; *ACO*, aconitase gene; *LDH*, lactate dehydrogenase gene; *ADE3,* cytoplasmic trifunctional C1-tetrahydrofolate (THF) synthase gene; *BNA2*, a putative tryptophan 2,3-dioxygenase or indoleamine 2,3-dioxygenase gene; *TES1*, peroxisomal acyl-CoA, thioesterase gene; MALE, maltose-binding protein gene; *MTT*, mitochondrial tricarboxylate transporter gene; *AMPD*, adenosine monophosphate deaminase gene; *POT1*, peroxisomal thiolase gene.

The industrial microbe *Yarrowia lipolytica* is an unconventional oleaginous yeast that is also classified by the US Food and Drug Administration as ‘generally regarded as safe’ (GRAS) (Zhao et al., 2021b). *Y. lipolytica* possesses unique physiological and metabolic features compared to the most widely used chassis strains *Escherichia coli* and *Saccharomyces cerevisiae*, which enhance its merits as a microbial cell factory (Liu et al., 2015). Firstly, *Y. lipolytica* has good tolerance for external environment stresses, such as low temperatures, high salt concentrations and acidic pH (Gonçalves et al., 2014). Secondly, the oleaginous yeast is able to utilize a myriad of carbon substrates for growth, including waste cooking oil (WCO) (Zinjarde, 2014; Pang et al., 2019; Li et al., 2022). This permits the valorization of waste streams and reduces the overall cost of production. Thirdly, *Y. lipolytica* is richly endowed with multiple pathways for the generation and accumulation of intracellular acetyl-CoA, which are important intermediaries of IA biosynthesis (Zhou et al., 2012; Ng et al., 2020). Finally, the yeast exhibits high tolerance for IA, thus allowing for accumulation of IA within (Zhao et al., 2019).

**FIGURE 1:**
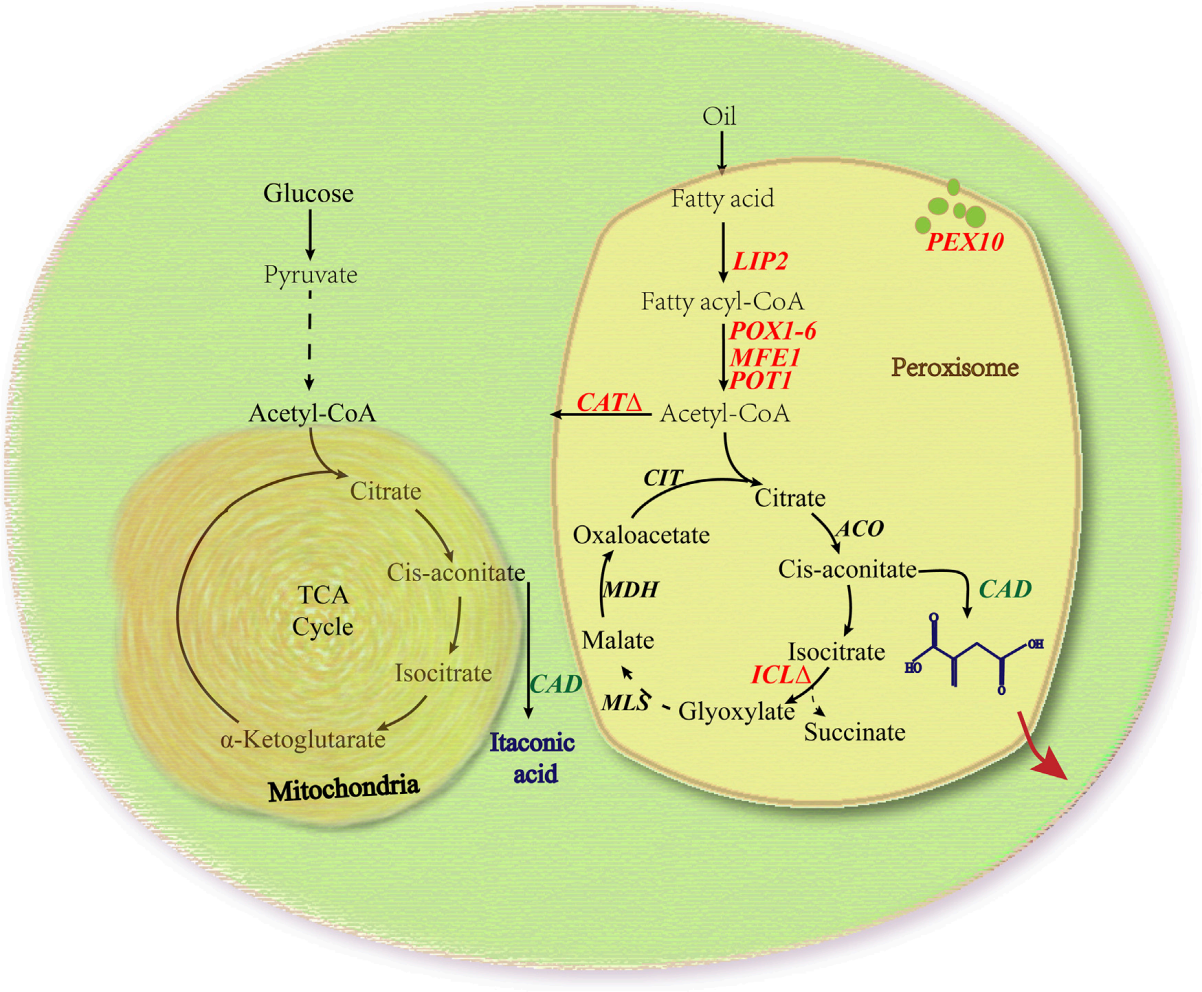
Simplified schematic of IA biosynthetic pathway in *Y. lipolytica*. Engineered *Y. lipolytica* uptakes and converts extracellular carbon sources such as glucose and waste cooking oil into IA products. Genes and metabolites of the native TCA and glyoxylate cycle pathway are identified in black, while heterologously introduced genes are shown in green and the endogenous genes used in this paper are shown in red. LIP2, lipases; POX1-6, six difffferent acyl-CoA oxidases; MFE1, multifunctional enzyme; POT1, peroxisomal thiolase; PEX10, a proteins required for peroxisome assembly; CAT, carnitine acetyltransferases; ICL, isocitrate lyase; CAD, iso-aconitic acid decarboxylase; ACO, aconitase; MLS, malate synthase; MDH, malate dehydrogenase; CIT, citrate synthase.

In our previous studies, we successfully engineered *Y. lipolytica* to produce limonene and bisabolene, where WCO was employed as the sole carbon source (Pang et al., 2019; Zhao et al., 2021b; Li et al., 2022). Motivated by earlier successes, we herein investigated the feasibility of producing IA from engineered *Y. lipolytica* on WCO (Figure 1). We expressed cis-aconitic acid decarboxylase (CAD) gene from *A. terreus* in either the cytosol or peroxisome of *Y. lipolytica* and assayed for production of IA in the extracellular supernatant. To further improve the final yield, the 10 genes involved in the production pathway of acetyl-CoA, an intermediate metabolite necessary for the synthesis of cis-aconitic acid, were each singly overexpressed. To minimize off-target flux channeling, we had also knocked out genes related to competing pathways in the peroxisome. Finally, IA titer up to 54.55 g/L was obtained in the engineered *Y. lipolytica* with a yield of 0.3 g/g WCO and a maximum productivity of 0.6 g/L/h without pH control in the 5 L bioreactor. At the time of writing, this is the highest titer of IA obtained with an engineered yeast cell factory.

## Materials and Methods

### Strains, Plasmids, Primers, and Cultivation Media

The *E. coli* strain DH5α was used as the host in this study for the cloning and plasmid construction. *E. coli* strains were routinely cultured at 37°C in Luria-Bertani (LB) media (1% tryptone, 0.5% yeast extract, and 1% sodium chloride contained) or on LB agar plates supplemented with 100 μg/ml of ampicillin. *Y. lipolytica* Po1g *KU70*Δ was used as the base strain in this study, which has been generated from the parental strain Po1g (a commonly used host strain for protein expression). This strain was used as it is known that the rate of precise homologous recombination (HR) increased substantially for deletion of the *KU70* gene in Po1g (Yu et al., 2016). Routine cultivation of *Y. lipolytica* strains was carried out at 30°C in YPD medium (1% yeast extract, 2% peptone and 2% dextrose contained) while the yeast synthetic complete medium (YNB) (0.67% yeast nitrogen base without amino acids, 2% glucose, 1.5% bacto agar) lacking the appropriate nutrients was used for the screening of transformants. The fermentation experiment used YPO medium containing WCO (1% yeast extract, 2% peptone, 1.18% WCO and 0.2% tween-80 contained), and the initial pH of cultivation media was 5.73. Among them, the amount of WCO added is calculated based on the same C atoms as glucose in YPD medium. The strains and plasmids used in this study are listed in Supplementary Table S1. The PCR primers used in this study were synthesized by Genewiz (Jiangsu, China) and are listed in Supplementary Table S2.

### Plasmid Construction

The *Y. lipolytica* expression vector pYLEX1 used in this study possesses the strong promoter hp4d, and its detailed information was provided in Li et al. (2021). Using primers CAD1-F/R and CAD2-F/R that were synthesized according to the existing sequence (GenBank ID: AB326105.1) in NCBI GenBank, two fragments of the *CAD* gene without introns were amplified from the *A. terreus* HAT418 genome and cloned into pYLEX1 to yield pYLEX1-CAD through adapted homologous recombination. The construction process of plasmid pYLEX1-CAD is depicted in Supplementary Figure S1. The sequences of the oligonucleotides used to amplify all the genes are listed in Supplementary Table S2 in the Additional file. Subsequently, the expression cassettes of other gene candidates were cloned into pYLEX1-CAD individually (Supplementary Figure S2). All recombinant plasmids were constructed using the One Step Cloning Kit from Vazyme Biotech Co., Ltd. (Nanjing, China). Transformants were plated on LB-ampicillin agar plates and incubated overnight at 37°C. Single colonies were inoculated into LB-ampicillin and cultured overnight at 37°C with shaking at 225 rpm. Plasmids were isolated, and the genes were verified by DNA sequencing.

Following that, all plasmids were linearized using the *Spe* Ⅰ enzyme and then transformed into the *Y. lipolytica* Po1g *KU70*Δ competent cells using lithium acetate/single-stranded vector DNA/polyethylene glycol method. The linearized plasmids introduced were integrated at the pBR322 locus of the strain Po1g *KU70*Δ. After 2 to 3 days of culture, the positive *Y. lipolytica* transformants were selected on YNB-LEU plates and subsequently confirmed by genomic DNA PCR analysis (Yu et al., 2016). Accordingly, in this study, the engineered *Y. lipolytica* Po1g *KU70*Δ strain was used as the host for all genetic modifications with gene knockouts and chromosomal expression constructs introduced *via* engineered pYLEX1 plasmids.

### Yeast Cultivation

Seed inoculum of *Y. lipolytica* were first cultured in a 20 ml tube with 5 ml YPD medium and incubated for 24 h in a shaking incubator set at 30°C and 220 rpm. Next, a 250 ml flask was filled with 50 ml YPO medium and inoculated at the seeding density of OD_600_ 0.1. The inoculated finished shake flasks were grown in a shaking incubator set at 30°C and 220 rpm. Fermented yeast cultures were collected on the fourth day and analyzed by GC-MS to determine and identify the IA content.

### Gene Knockout

The *ICL*Δ strain was generated by knocking out the ORF region gene of *ICL via* the homologous recombination (HR) mechanism, which replaced *ICL* with the hygromycin B resistance marker gene (*HPH*) amplified from pSH69-Hph using the primer pairs ICL-Hph-F/R. To this end, two targeting arms (upstream and downstream flanking sequences of *ICL*), each approximately 1,000 bp in length, were amplified using PCR from the genomic DNA of Po1g-2G and ligated to the 5′ and 3′ ends of the *HPH* gene, respectively. After transformation of the *ICL* disruption cassette into *Y. lipolytica* cells, a gene replacement event occurs *via* double-crossover homologous recombination within the two flanking homology arms at the targeted locus. Transformants were grown in the YPDH solid medium (30°C, under dark conditions) supplemented with hygromycin and chosen randomly. The correct *ICL*Δ strain was confirmed by PCR with ICL-Hph-knock-F and ICL-Hph-knock-R primers. The construction of the *CAT*Δ strain was carried out using a similar procedure.

### Visualizing Fluorescence Distribution by Laser Scanning Confocal Microscopy

To test the peroxisomal targeting ability of enhanced peroxisome targeting signal ePTS1, yeast cells expressing *hrGFPO-ePTS1* were cultured in 50 ml YPD medium for 24 h. For simultaneous visualization of hrGFPO and Nile red, precultures incubated in 50 ml YPD were stained by adding Nile red solution (1 mg/ml) in acetone to the cell suspension (0.1 v/v) and incubated for 60 min in the dark at room temperature. The stained cells were washed with normal saline and resuspended in potassium phosphate buffer (pH 7.4) before being transferred onto glass slides to visualize hrGFPO at 488 nm and Nile red at 561 nm with an Olympus FV1000 confocal laser scanning microscope.

### Esterification of the Fermented Supernatant

2 ml of the fermented supernatant was added to 1.5 ml of 10% HCl-CH_3_OH solution, which was esterified at 62°C for 3 h. Then, 2 ml of n-hexane was added and the resultant mixture was violently shaken for 1 min to dissolve the dimethyl itaconate. After centrifugation (6,000 rpm, 5 min), the upper organic phase was transferred into another clean bottle for detection.

### GC-MS Analysis

0.6 μl of the upper organic phase from the above Section was analyzed by GC-MS using an Agilent 7890A GC with a 5975C MSD equipped with an HP-5MS column (30 m × 0.25 mm × 0.25 μm, Agilent, Santa Clara, CA, United States). The GC oven temperature was initially held at 60°C for 2 min, and then ramped up to 250°C at a rate of 10°C/min and held for 9 min. The split ratio was 10:1. Helium was used as the carrier gas, with an inlet pressure of 13.8 psi. The injector was maintained at 250°C and the ion source temperature was set to 220°C. The final data analysis was performed using the Enhanced Data Analysis software (Agilent, Santa Clara, CA, United States) to obtain the standard curve of dimethyl itaconate, and the area obtained after the sample is analyzed and detected by the instrument is brought into the formula of the standard curve to obtain the output of dimethyl itaconate. The titer of IA is obtained by converting with the esterification rate obtained in the above section.

### Statistical Analysis

Differences in titers between the control strain and other strains were evaluated using SPSS 22.0 software for Windows (SPSS, Chicago, IL, United States). One-way ANOVA analyses were carried out with a confidence interval of 95% and statistical significance between the groups and the relevant control was considered if *p*-value < 0.05.

### Bioreactor Fermentations

Bioreactor fermentation was batched processed using an optimal medium formulation containing 59 g/L WCO, 16 g/L yeast extract, 8 g/L peptone and 10 g/L tween-80. The strain was first seeded in 50 ml YPD medium in 250 ml shake flasks, cultured at 30°C and 220 rpm for 16 h. Following that, the bioreactor containing 3 L of YPO medium were inoculated with the seed cultures at an OD_600_ of 1.

Fermentation without any pH control was carried out in a 5 L stirred fermenter (Shanghai Baoxing Bioengineering Equipment Co., Ltd., Shanghai, China) at 30°C and 1 vvm. The bioreactor pressure was maintained at 0.06 MPa. The impeller stirring speed was 400 rpm.

## Results and Discussion

### Heterologous Expression of *A. terreus* Cis-Aconitic Acid Decarboxylase in *Y. lipolytica*

**FIGURE 2:**
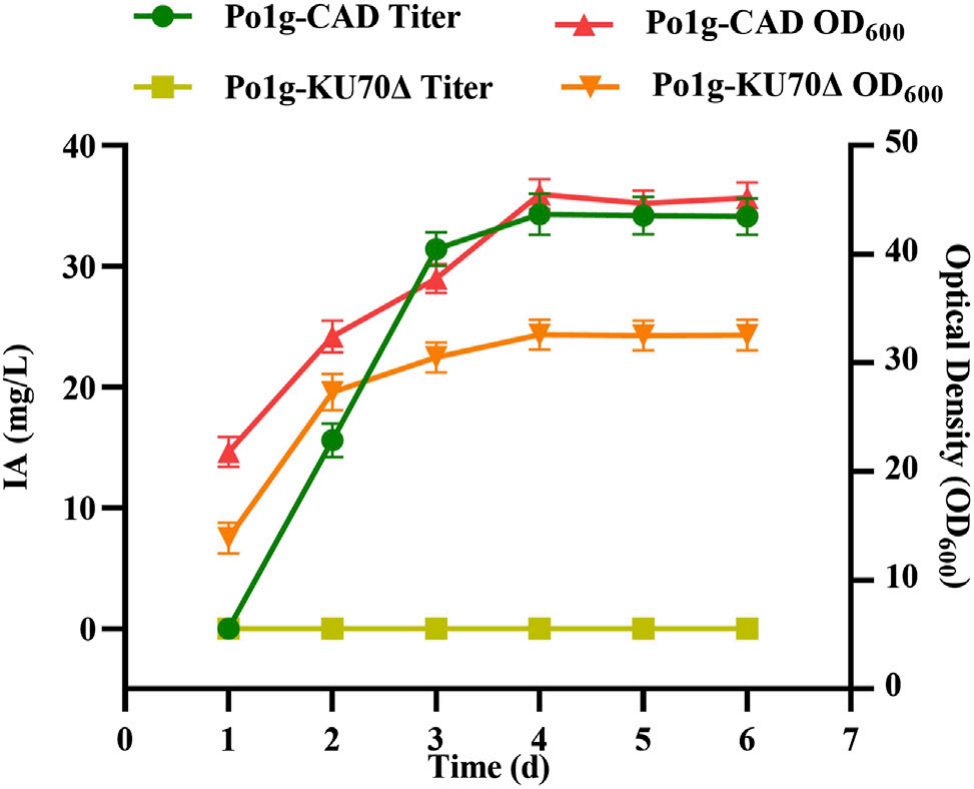
IA production in *Y. lipolytica* strains expressing the *CAD* gene. The titer of IA and biomass of *Y. lipolytica* were determined by shaking flask fermentation of Po1g-CAD strain and control strain Po1g in YPO culture. All values presented are the mean of three biological replicates ± standard deviation.

In *A. terreus*, IA is generated from the decarboxylation of the TCA intermediate cis-aconitic acid by the CAD enzyme (Bonnarme et al., 1995; Tevz et al., 2010). To test if *A. terrus’s CAD* gene can be expressed successfully in *Y. lipolytica* without codon optimization, we first cloned the associated gene from *A. terreus* HAT418 strain into *Y. lipolytica* strain Po1g *KU70*Δ, with the gene’s intron spliced out. In the gene sequencing analysis that followed, we discovered that the actual PCR-amplified gene sequence was different from the genome sequence shown in NCBI database. Our sequence data for *A. terreus* HAT418 *CAD* gene was submitted to GenBank under the accession number MT862134.1. Overexpression of the *CAD* gene in *Y. lipolytica* Po1g *KU70*Δ resulted in the creation of strain Po1g-CAD. We subjected both the engineered strain with cytosolic CAD and control strain without CAD to shake flask fermentation and assayed for IA continuously over a period of 6 days. We confirmed that IA was produced only in the engineered *Y. lipolytica* but not in its wild type. IA levels were first detected in the supernatant on day 2 and they increased gradually with time until a maximum yield of 33.12 mg/L was obtained on day 4 (Figure 2). This is contrasted with the Po1g *KU70*Δ original strain where no IA production detected, thereby confirming that the heterologous expression of the *A. terreus* CAD is necessary for IA production in *Y. lipolytica*.

### Peroxisomal Targeting of Heterologous Cis-Aconitic Acid Decarboxylase Gene Improved Itaconic Acid Production

β-oxidation of long chain fatty acids in eukaryotes are known to occur mainly in the peroxisomes (Wache et al., 2001; Hanko et al., 2018). In *Y. lipolytica,* this process produces acetyl-CoA which then enters the glyoxylate cycle for synthesis of the IA precursor, cis-aconitic acid (Dominguez et al., 2010; Koivistoinen et al., 2013; Xu et al., 2017). Several studies have shown that subcellular localization of specific enzymes or metabolic pathways not only increase product conversion efficiency, but is also able to suppress the undesirable effects of competitive metabolic inhibition (Zhu et al., 2018; Yang et al., 2019; Zhu et al., 2021). As such, this approach of subcellular compartmentalization is adopted in our study and complemented with the use of WCO as the substrate to enable sustainable, efficient and low-cost production of IA. To this end, IA production from the glyoxylate cycle in *Y. lipolytica* was ensured by targeting the involved heterologous enzymes to the peroxisomal matrix through the addition of enhanced peroxisomal targeting signal (ePTS1) after its gene sequence. The ePTS1 applied in this instance has been shown to be localized in *S. cerevisiae* (DeLoache et al., 2016).

**FIGURE 3:**
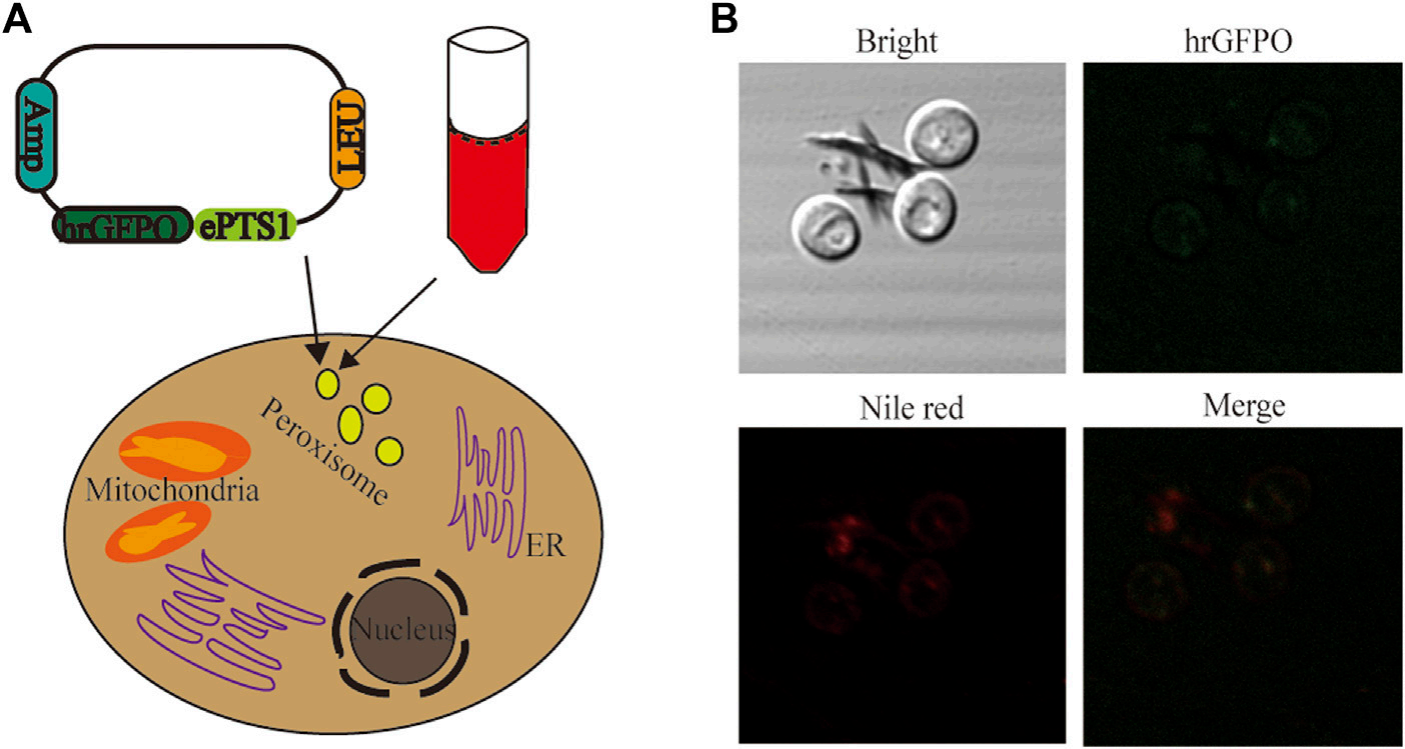
Investigation of the localization of peroxisomes. **(A)** Schematic diagram of experimental design. hrGFPO-ePTS1 is used to specififically mark peroxisomes in *Y. lipolytica*. Nile red is used to show intracellular peroxisomes regions. **(B)** Localization observation of peroxisomes use the Nile red and strain Po1g-hrGFPO-ePTS1 through LSCM.

Two separate dyes, Nile red and green fluorescence, were employed for staining of the yeast cells to validate the peroxisomal targeting ability of ePTS1. In an earlier study, it was shown that hrGFPO, encoding the green fluorescence protein, was most strongly expressed in Po1g *KU70*Δ (Zhao et al., 2021a). The plasmid with sequence ePTS1 added after the hrGFPO protein sequence was retransformed into yeast, resulting in strain Po1g-hrGFPO-ePTS1 (Figure 3A). Nile red fluorescence, on the other hand, was used to stain the peroxisomes of the yeast cells. To determine if ePTS1 could be successfully localized to peroxisomes in *Y. lipolytica*, Laser Scanning Confocal Microscopy (LSCM) was performed to observe the location of the two different fluorescence in yeast cells. As shown in Figure 3B, a green fluorescent protein with localization signal ePTS1, which exhibits green light under microscope irradiation, was expressed in the engineered yeast. Yeast cells after Nile red staining also show localized red fluorescence under the microscope. Combining these two images, we observed that the green and red shades overlap almost completely and produce a bright yellow light. Therefore, it can be confirmed that ePTS1 plays a role in determining the location of the peroxisome could be used as a peroxisomal targeting sequence for *Y. lipolytica*.

**FIGURE 4:**
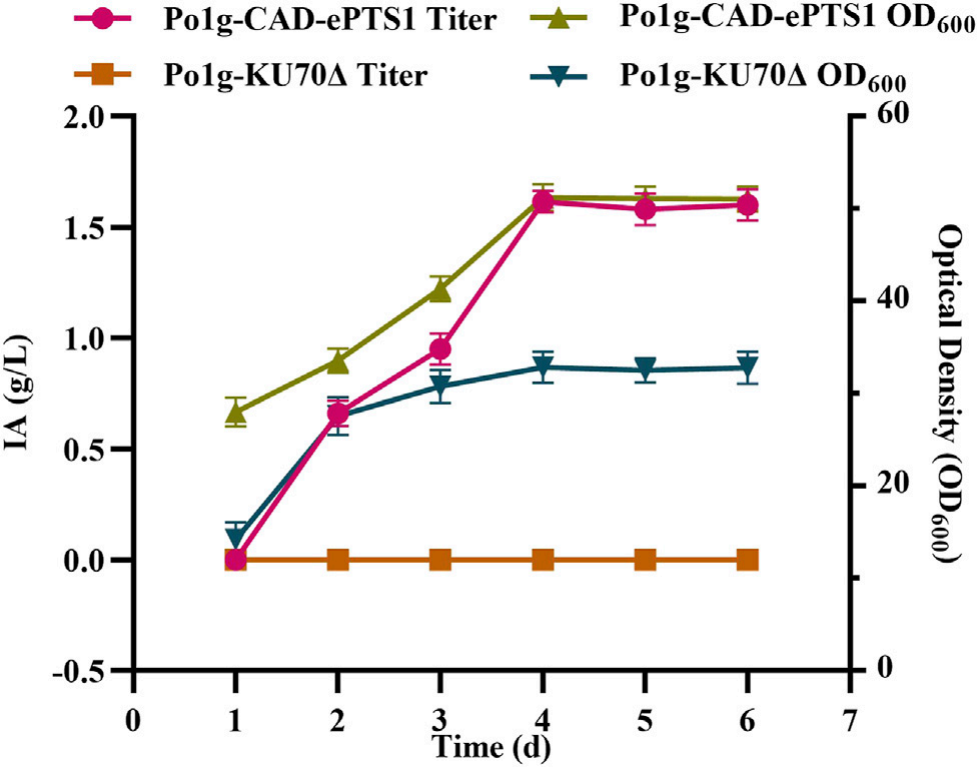
IA production in *Y. lipolytica* strains expressing the CAD-ePTS1 gene. The titer of IA and biomass were determined by shaking flask fermentation of Po1g-CAD-ePTS1 strain and control strain Po1g in YPO culture. All values presented are the mean of three biological replicates ± SD.

Subsequently, the plasmid pYLEX1-CAD-ePTS1 constructed through the ligation of ePTS1 downstream of the *CAD* gene was integrated into the *Y. lipolytica* Po1g *KU70*Δ chromosomes of the strain. The resulting engineered strain was cultured in the YPO medium and the 6 days course of IA production titers and biomass were shown in Figure 4. The titers of IA increased continuously from the beginning of cultivation up to day 4 with the highest titer having reached 1.58 g/L. Following this, the titers of IA gradually stabilized, likely owing to WCO depletion. Notably, we also compared the use of WCO and glucose in this subcellular compartmentalized approach to generate IA under the same conditions. The use of WCO had resulted in almost 100-folds increase in IA titer as compared to glucose (13.68 mg/L of IA) as the carbon source, hence implying that WCO was superior to glucose for IA production in these conditions. We also observed that the overproduction of IA has a positive effect on the cell growth. Together, our results demonstrate that the expression and localization of *CAD* in the peroxisomes of *Y. lipolytica* can lead to substantial increase in IA production.

### Overexpression of Endogenous Genes Involved in the Acetyl-CoA Production Pathway of *Y. lipolytica*

**FIGURE 5:**
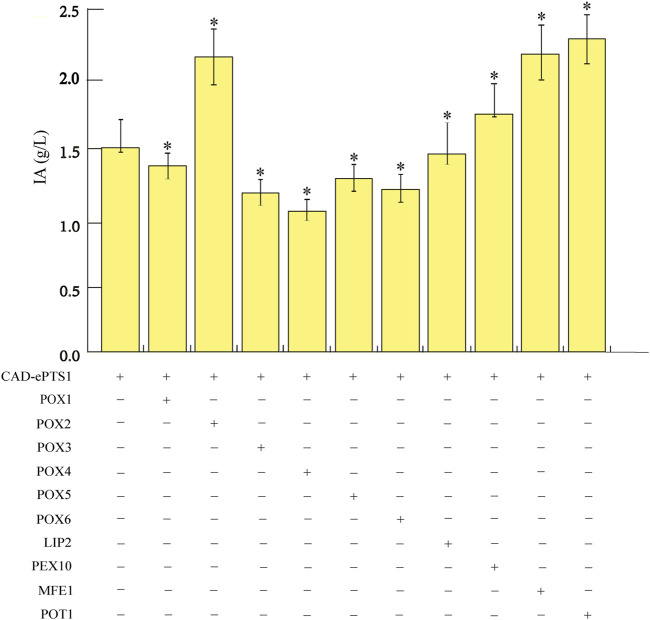
Effects of overexpressing genes involved in the acetyl-CoA production pathway on IA production. The genes involved in the acetyl-CoA production pathway, consisting of *LIP2, POX1-6, MFE1, POT1* and *PEX10,* were overexpressed individually. Titers of IA produced by the strains were quantified after 6 days of cultivation in shake flasks with YPO medium. All values presented are the mean of three biological replicates ± standard deviation. *p<0.05, significantly different from control by ANOVA.

To further enhance IA production in *Y. lipolytica*, we attempted to study the pathway genes involved in the conversion of oils to fatty acids and the utilization of fatty acids to raise the flux of precursor acetyl-CoA. The β-oxidation of fatty acids is a four-reaction cycle comprising of oxidation, hydration, dehydrogenation, and thiolysis, which results in one molecule of acetyl-CoA released in the peroxisome (Braga and Belo, 2016). In *Y. lipolytica*, the first step of fatty acid β-oxidation can be catalyzed by six different acyl-CoA oxidases (POX1-6) (Beopoulos et al., 2008). The second and third steps of β-oxidation are catalyzed by a multifunctional enzyme (MFE1) (Black et al., 2000; Dulermo et al., 2013), and the final step is catalyzed by peroxisomal thiolase (POT1) (Wang et al., 2020). As such, the genes involved in the β-oxidation pathway were overexpressed in an attempt to increase the flux towards IA. Ten genes, consisting of *LIP2* (encoding lipases, Zhang et al., 2021b), *POX1-6* (Ledesma-Amaro and Nicaud, 2016), *MFE1* (Haddouche et al., 2010), *POT1* (Smith et al., 2000), and *PEX10* (encoding a proteins required for peroxisome assembly, Zhang et al., 2021b), were overexpressed individually and investigated for their effects on IA overproduction to determine the genes critical for IA biosynthesis in the acetyl-CoA production pathway. To this end, ten strains were constructed on the basis of the strain expressing *CAD-ePTS1* gene, including the ten endogenous genes in the acetyl-CoA production pathway of *Y. lipolytica*; all genes were integrated into the chromosomes of *Y. lipolytica* Po1g *KU70*Δ. These ten engineered strains were then cultured in YPO medium for 6 days in shake flasks. The IA titers of the strains showed that the overexpression of the individual corresponding genes could improve IA production compared to the control strain expressing only the respective *CAD-ePTS1* gene. Among them, the POT1-overexpressed strain (hereafter named Po1g-2G), achieved the highest titers of 2.42 g/L for IA after 4 days of cultivation (Figure 5). The results indicated that overexpression of this key enzymes can effectively promote the fatty acid degradation process and release the most acetyl-CoA molecules for IA biosynthesis. This observation is consistent with several other studies in which POT1 has already been demonstrated to be the key rate-limiting enzyme in the β-oxidation pathway (Ma et al., 2020; Zhang et al., 2021b). Therefore, the engineered strain Po1g-2G was used for subsequent engineering efforts to boost IA production.

### Effects of Deletion of the Carnitine Acetyltransferases and Isocitrate Lyase Genes on Itaconic Acid Production in *Y. lipolytica*

The yield of IA can be further improved by reducing the loss of the precursor acetyl-CoA and preventing the synthesis of cis-aconitic acid from the glyoxylate cycle into downstream products such as succinic acid. The carnitine acetyltransferases (CAT) is responsible for transporting acetyl-CoA between different organelles, which can reversibly link the acetyl units to the carrier molecule carnitine (Strijbis et al., 2008; Strijbis et al., 2010). Meanwhile, the isocitrate lyase (ICL) manages the conversion of isocitrate into succinic acid and glyoxylic acid (Koivistoinen et al., 2013). To verify if either of these enzymes assume a major role in IA production, the corresponding genes singly were deleted from Po1g-2G, resulting in the creation of strains Po1g-2G-CATΔ and Po1g-2G-ICLΔ. After cultivating the resulting strains in shake flasks in YPO medium, it was found that higher IA production reaching 3.33 g/L was observed in ICL knockout strain as compared to CAT knockout strain with 2.8 g/L titers. This suggests that blocking the downstream pathway improves IA production while blocking the efflux effect of the acetyl coenzyme in the peroxisome is not as advantageous. Therefore, the Po1g-2G-ICLΔ strain was selected as the final optimized strain.

### Itaconic Acid Production by the Engineered *Y. lipolytica* in Bioreactor

**FIGURE 6:**
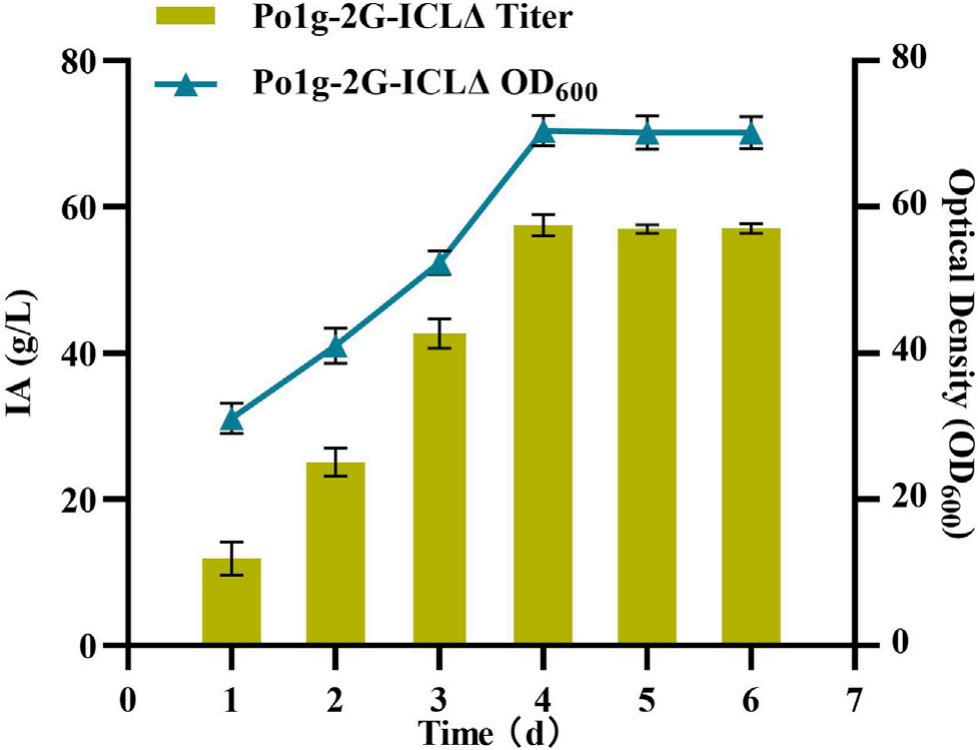
IA production in bioreactor of *Y. lipolytica* strain expressing the 2G-ICLΔ gene. The titer of IA and biomass were determined by bioreactor fermentation of Po1g-2G-ICLΔ strain in YPO culture. All values presented are the mean of three biological replicates ± SD.

One of the most crucial issues in platform chemicals production is in achieving a high product titers consistently (Gao et al., 2016; Li et al., 2020). To investigate the performance of IA-producing *Y. lipolytica* at conditions that are more relevant for large-scale application, a 5 L bioreactor was employed. Unlike the procedure conducted in the shaking flask fermentation method, here, the composition of the growth medium was altered and the approach of adding sufficient WCO substrate at once was adopted to avoid the problems caused by fed batch fermentation. In the phase of active cell growth between 24 and 96 h, the Po1g-2G-ICLΔ strain intensively produced IA. During this period, the average specific rate of IA synthesis was 0.8 g/L/h, and the maximum specific rate of 2.3 g/L/h was observed between the 76–96-h intervals (Figure 6). Hence, the maximum titer of IA was 54.55 g/L after the 96-h reaction in the fermenter. At the time of writing, this is the highest IA production achieved by a yeast host reported worldwide. As such, *Y. lipolytica* would be a promising industrial host for IA production from renewable feedstock. Our study also demonstrated that the circular bioeconomy concept can be an effective model for scale-up production of valuable biochemical, in particular with the valorization of WCO as raw material.

## Conclusion

With increasing global interest in environmental protection and sustainable development, the use of low-cost waste to produce valuable platform chemicals in the industrial scale is gaining attention. In the few studies conducted to date, IA production in engineered strains of *Y. lipolytica* was predominantly using glucose as the primary carbon source (Blazeck et al., 2015; Zhao et al., 2019). Even so, production titers had remained suboptimal (Table 1). This had limited the feasibility of large-scale industrial adoption. Here, we employed the cheap raw material WCO to increase acetyl-CoA availability for conversion into IA in the peroxisome of *Y. lipolytica*. By applying both systems metabolic engineering and bioprocessing optimization strategies in unison, we achieved IA titers of 3.33 g/L in shake flasks and up to 54.55 g/L in stirred-tank bioreactor on WCO as the carbon source without the need for pH control. This amounted to more than 34-folds as compared to the initial titers of 1.58 g/L IA before the optimization of strain and fermentation conditions. In this study, as the supernatant may contain WCO that was not consumed completely, IA cannot be detected directly by HPLC. We used esterification of the supernatant to detect the yield of dimethyl itaconate. While this method, in principle, can be used to determine the theoretical final yield of IA from the esterification rate, it is not the best approach to quantify the exact yield of IA. The development of a more robust and higher throughput method of analysis should be considered in future studies. Furthermore, the yield of organic acids produced by *Y. lipolytica* is primarily affected by the genetic mechanism and various environmental factors, such as the carbon source, nitrogen source, temperature, pH, iron concentration, and dissolved oxygen levels. As such, since bioreactor fermentation with WCO as the sole carbon source is still relatively understudied, further optimization of the fermentation conditions could improve IA yields. Nonetheless, the present work on the production of IA by WCO still provides valuable insights that will facilitate further efforts in the biosynthesis of this compound. The results obtained suggest that the oleaginous yeast *Y. lipolytica* is an attractive platform as it provides a viable and scalable pathway to the overproduction of IA and most notably, one that is sustained by waste conversion. However, given the extensive knowledge on IA gene regulation and fermentation conditions, it is believed that higher productivities of IA can be achieved through further engineering of this yeast strain and the optimization of fermentation conditions in subsequent studies.

## Data Availability

The datasets presented in this study can be found in online repositories. The names of the repository/repositories and accession number(s) can be found in the article/Supplementary Material.
