# Supplementary material for: Engineering Yarrowia lipolytica to Produce Itaconic Acid From Waste Cooking Oil
Source: Front Bioeng Biotechnol. 2022 Apr 25;10:888869. doi: 10.3389/fbioe.2022.888869 (PMC9083544; doi:10.3389/fbioe.2022.888869)
Supplement: Supplementary file 1 [file DataSheet1.docx]

**SUPPLEMENTARY INFORMATION**

**Engineering *Yarrowia lipolytica* to produce itaconic acid from waste cooking oil**

Lanxin Rong^1^, Lin Miao^1^, Shuhui Wang^1^, Yaping Wang^1^, Shiqi Liu^1^, Zhihui Lu^1^, Baixiang Zhao^1^, Cuiying Zhang^1^, Dongguang Xiao^1^, Krithi Pushpanathan^2^, Adison Wong^2**^, Aiqun Yu^1*^

**Table S1** **Plasmids and strains used in this study**

| **Plasmids** | **Features** | **Reference** |
| --- | --- | --- |
| pYLEX1 | *Y. lipolytica*-integrative plasmid, Php4d-TXPR2, LEU2 | Li, 2021 |
| pYLEX1-CAD | P_hp4d_-CAD-T_XPR2_, LEU2 | This study |
| pYLEX1-hrGFPO-ePTS1 | P_hp4d_-hrGFPO-ePTS1-T_XPR2_, LEU2 | This study |
| pYLEX1-CAD-ePTS1 | P_hp4d_-CAD-ePTS1-T_XPR2_, LEU2 | This study |
| pYLEX1-LIP | P_hp4d_-LIP-T_XPR2_, LEU2 | This study |
| pYLEX1-CAD-ePTS1-LIP | P_hp4d_-CAD-ePTS1-T_XPR2_, P_hp4d_-LIP-T_XPR2_, LEU2 | This study |
| pYLEX1-POX1 | P_hp4d_-POX1-T_XPR2_, LEU2 | This study |
| pYLEX1-CAD-ePTS1-POX1 | P_hp4d_-CAD-ePTS1-T_XPR2_, P_hp4d_-POX1-T_XPR2_, LEU2 | This study |
| pYLEX1-POX2 | P_hp4d_-POX2-T_XPR2_, LEU2 | This study |
| pYLEX1-CAD-ePTS1-POX2 | P_hp4d_-CAD-ePTS1-T_XPR2_, P_hp4d_-POX2-T_XPR2_, LEU2 | This study |
| pYLEX1-POX3 | P_hp4d_-POX3-T_XPR2_, LEU2 | This study |
| pYLEX1-CAD-ePTS1-POX3 | P_hp4d_-CAD-ePTS1-T_XPR2_, P_hp4d_-POX3-T_XPR2_, LEU2 | This study |
| pYLEX1-POX4 | P_hp4d_-POX4-T_XPR2_, LEU2 | This study |
| pYLEX1-CAD-ePTS1-POX4 | P_hp4d_-CAD-ePTS1-T_XPR2_, P_hp4d_-POX4-T_XPR2_, LEU2 | This study |
| pYLEX1-POX5 | P_hp4d_-POX5-T_XPR2_, LEU2 | This study |
| pYLEX1-CAD-ePTS1-POX5 | P_hp4d_-CAD-ePTS1-T_XPR2_, P_hp4d_-POX5-T_XPR2_, LEU2 | This study |
| pYLEX1-POX6 | P_hp4d_-POX6-T_XPR2_, LEU2 | This study |
| pYLEX1-CAD-ePTS1-POX6 | P_hp4d_-CAD-ePTS1-T_XPR2_, P_hp4d_-POX6-T_XPR2_, LEU2 | This study |
| pYLEX1-MFE1 | P_hp4d_-MFE1-T_XPR2_, LEU2 | This study |
| pYLEX1-CAD-ePTS1-MFE1 | P_hp4d_-CAD-ePTS1-T_XPR2_, P_hp4d_-MFE1-T_XPR2_, LEU2 | This study |
| pYLEX1-POT1 | P_hp4d_-POT1-T_XPR2_, LEU2 | This study |
| pYLEX1-CAD-ePTS1-POT1 | P_hp4d_-CAD-ePTS1-T_XPR2_, P_hp4d_-POT1-T_XPR2_, LEU2 | This study |
| pYLEX1-PEX10 | P_hp4d_-PEX10-T_XPR2_, LEU2 | This study |
| pYLEX1-CAD-ePTS1-PEX10 | P_hp4d_-CAD-ePTS1-T_XPR2_, P_hp4d_-PEX10-T_XPR2_, LEU2 | This study |
| **Strains** | **Genotype** | **Reference** |
| Po1g KU70Δ | MatA, leu2-270, ura3-302::URA3, xpr2-332, axp-2, ku70- | Li jian., 2021 |
| Po1g-CAD | MatA, leu2-270, ura3-302::URA3, xpr2-332, axp-2, ku70-, CAD | This study |
| Po1g-hrGFPO-ePTS1 | MatA, leu2-270, ura3-302::URA3, xpr2-332, axp-2, ku70-, hrGFPO-ePTS1 | This study |
| Po1g-CAD-ePTS1 | MatA, leu2-270, ura3-302::URA3, xpr2-332, axp-2, ku70-, CAD-ePTS1 | This study |
| Po1g-CAD-ePTS1-LIP | MatA, leu2-270, ura3-302::URA3, xpr2-332, axp-2, ku70-, CAD-ePTS1-LIP | This study |
| Po1g-CAD-ePTS1-POX1 | MatA, leu2-270, ura3-302::URA3, xpr2-332, axp-2, ku70-, CAD-ePTS1-POX1 | This study |
| Po1g-CAD-ePTS1-POX2 | MatA, leu2-270, ura3-302::URA3, xpr2-332, axp-2, ku70-, CAD-ePTS1-POX2 | This study |
| Po1g-CAD-ePTS1-POX3 | MatA, leu2-270, ura3-302::URA3, xpr2-332, axp-2, ku70-, CAD-ePTS1-POX3 | This study |
| Po1g-CAD-ePTS1-POX4 | MatA, leu2-270, ura3-302::URA3, xpr2-332, axp-2, ku70-, CAD-ePTS1-POX4 | This study |
| Po1g-CAD-ePTS1-POX5 | MatA, leu2-270, ura3-302::URA3, xpr2-332, axp-2, ku70-, CAD-ePTS1-POX5 | This study |
| Po1g-CAD-ePTS1-POX6 | MatA, leu2-270, ura3-302::URA3, xpr2-332, axp-2, ku70-, CAD-ePTS1-POX6 | This study |
| Po1g-CAD-ePTS1-MFE1 | MatA, leu2-270, ura3-302::URA3, xpr2-332, axp-2, ku70-, CAD-ePTS1-MFE1 | This study |
| Po1g-CAD-ePTS1-POT1 | MatA, leu2-270, ura3-302::URA3, xpr2-332, axp-2, ku70-, CAD-ePTS1-POT1 | This study |
| Po1g-CAD-ePTS1-PEX10 | MatA, leu2-270, ura3-302::URA3, xpr2-332, axp-2, ku70-, CAD-ePTS1-PEX10 | This study |
| Po1g-2G-ICLΔ | MatA, leu2-270, ura3-302::URA3, xpr2-332, axp-2, ku70-, CAD-ePTS1- POT1-ICLΔ-hph | This study |
| Po1g-2G-CATΔ | MatA, leu2-270, ura3-302::URA3, xpr2-332, axp-2, ku70-, CAD-ePTS1- POT1-CATΔ-hph | This study |

**Table S2 Primers used in PCR**

| Applications | Primers | Sequences |
| --- | --- | --- |
| Amplify *CAD-1* gene from *A. terreus* genomic DNA, forward primer. | CAD1-F | acaaccacacacatccacaATGACCAAACAATCTGCGGACA |
| Amplify *CAD-1* gene from *A. terreus* genomic DNA, reverse primer. | CAD1-R | TGCTGCAACAGGCCCCAGTTTCTGTCCATATCCAATCACCCTGC |
| Amplify *CAD-2* gene from *A. terreus* genomic DNA, forward primer. | CAD2-F | GCAGGGTGATTGGATATGGACAGAAACTGGGGCCTGTTGCAGCA |
| Amplify *CAD-2* gene from *A. terreus* genomic DNA, reverse primer. | CAD2-R | ttagtttcgggttcccacgtgTTATACCAGTGGCGATTTCACG |
| Amplify *hrGFPO-ePTS1* gene from pYLEX1-hrGFPO, forward primer.. | GFP-ePTS1-F | acaaccacacacatccacAATGGTGTCTAAGCAGATCCTGAAGA |
| Amplify *hrGFPO-ePTS1* gene from pYLEX1-hrGFPO, reverse primer | GFP-ePTS1-R | ttagtttcgggttcccagcttggatcgtcgtcctcggcccagcacgtgGTGGTGGTGGTGGTGGTGC |
| Amplify *CAD-ePTS1* gene from pYLEX1-CAD, reverse primer | CAD-ePTS1-R | ttagtttcgggttcccacgtgTTAcagcttggatcgtcgtcctcggcccagTACCAGTGGCGATTTCACG |
| Amplify *LIP* gene form *Y. lipolytica* genomic DNA, forward primer | LIP-F | acaaccacacacatccacaATGGTCAGCTTTGGAGCTCG |
| Amplify *LIP* gene form *Y. lipolytica* genomic DNA, reverse primer | LIP-R | ttagtttcgggttcccacgtgTTAGTTGGAGAGCTCGAGACCC |
| Amplify *POX1* gene form *Y. lipolytica* genomic DNA, forward primer | POX1-F | acaaccacacacatccacAATGGCCAAGGAGCGAGGT |
| Amplify *POX1* gene form *Y. lipolytica* genomic DNA, reverse primer | POX1-R | ttagtttcgggttcccacgtgTCACTCATCGAGATCGCAAATTT |
| Amplify *POX2* gene form *Y. lipolytica* genomic DNA, forward primer | POX2-F | acaaccacacacatccacAATGAACCCCAACAACACTGGC |
| Amplify *POX2* gene form *Y. lipolytica* genomic DNA, reverse primer | POX2-R | ttagtttcgggttcccacgtgCTATTCCTCATCAAGCTCGCAA |
| Amplify *POX3* gene form *Y. lipolytica* genomic DNA, forward primer | POX3-F | acaaccacacacatccacAATGATCTCCCCCAACCTCACA |
| Amplify *POX3* gene form *Y. lipolytica* genomic DNA, reverse primer | POX3-R | ttagtttcgggttcccacgtgCTATTCCTCGTCCAGCTCGCA |
| Amplify *POX4* gene form *Y. lipolytica* genomic DNA, forward primer | POX4-F | acaaccacacacatccacAATGATCACCCCAAACCCCG |
| Amplify *POX4* gene form *Y. lipolytica* genomic DNA, reverse primer | POX4-R | ttagtttcgggttcccacgtgTTACTGAATATCCTCGGGCTCC |
| Amplify *POX5* gene form *Y. lipolytica* genomic DNA, forward primer | POX5-F | acaaccacacacatccacAATGAACAACAACCCCACCAACG |
| Amplify *POX5* gene form *Y. lipolytica* genomic DNA, reverse primer | POX5-R | ttagtttcgggttcccacgtgCTACTCGTCCAGGTCGCAAATC |
| Amplify *POX6* gene form *Y. lipolytica* genomic DNA, forward primer | POX6-F | actttggtctactccggtacAATGCTCTCTCAACAGTCCCTCAA |
| Amplify *POX6* gene form *Y. lipolytica* genomic DNA, reverse primer | POX6-R | gggacaggccatggaggtaccCTACTCATCCTCAAGAGAGCAAATTT |
| Amplify *MFE1* gene form *Y. lipolytica* genomic DNA, forward primer | MFE1-F | actttggtctactccggtacAATGTCTGGAGAACTAAGATACGACGG |
| Amplify *MFE1* gene form *Y. lipolytica* genomic DNA, reverse primer | MFE1-R | gggacaggccatggaggtaccTTAGAGCTTAGCATCCTTGGGG |
| Amplify *POT1* gene form *Y. lipolytica* genomic DNA, forward primer | POT1-F | acaaccacacacatccacAATGGACCGACTTAACAACCTCG |
| Amplify *POT1* gene form *Y. lipolytica* genomic DNA, reverse primer | POT1-R | ttagtttcgggttcccacgtgTTACTCGGCAACAACCAGAGAA |
| Amplify *PEX10* gene form *Y. lipolytica* genomic DNA, forward primer | PEX10-F | actttggtctactccggtacAATGGACTACTTTTCGTCACTCAACG |
| Amplify *PEX10* gene form *Y. lipolytica* genomic DNA, reverse primer | PEX10-R | gggacaggccatggaggtaccTTACACCATCAGTCGTCTCAGACC |
| **Co-overexpression (general primer)** | | |
| Amplify the second gene expression cassette, forward primer | CAD-ePTS1-LIP-F | ccatccagcctcgcgtcgcCCCGCGCCCACCGGAAG |
| Amplify the second gene expression cassette, reverse primer | CAD-ePTS1-LIP-R | acgtcttgctggcgttcgcgaCATGAGAATTCGGACACGGG |
| **Gene knockout** | | |
| Amplify deletion cassette for upstream *ICL* gene from *Y. lipolytica*, forward primer. | ICL-up-F | ccttttgccagtatatcca |
| Amplify deletion cassette for upstream *ICL* gene from *Y. lipolytica*, reverse primer. | ICL-up-R | agggtattctgggcctccatgtcttttgtatgcttggtcagtcta |
| Amplify deletion cassette for downstream *ICL* gene from *Y. lipolytica*, forward primer. | ICL-down-F | atgtgaatgctggtcgctatactggcagtttgtttagcaaaatatatt |
| Amplify deletion cassette for downstream *ICL* gene from *Y. lipolytica*, reverse primer. | ICL-down-R | agtaggttgtctggcttttcct |
| Amplify deletion cassette for *hph* gene from pSH69, forward primer. | ICL-hph-F | tagactgaccaagcatacaaaagacatggaggcccagaataccct |
| Amplify deletion cassette for *hph* gene from pSH69, reverse primer. | ICL-hph-R | aatatattttgctaaacaaactgccagtatagcgaccagcattcacat |
| Amplify deletion cassette for upstream *CAT* gene from *Y. lipolytica*, forward primer. | CAT-up-F | atataccgaggcatgcaatttgat |
| Amplify deletion cassette for upstream *CAT* gene from *Y. lipolytica*, reverse primer. | CAT-up-R | caaggagggtattctgggcctccatgtcggtgaaagcgcgggtagacgtgagtcgagc |
| Amplify deletion cassette for downstream *CAT* gene from *Y. lipolytica*, forward primer. | CAT-down-F | gtatgtgaatgctggtcgctatactgatgcggttaaaagttcaagtaaaataatgat |
| Amplify deletion cassette for downstream *CAT* gene from *Y. lipolytica*, reverse primer. | CAT-down-R | gacgagcatctcgaatcgaag |
| Amplify deletion cassette for *hph* gene from pSH69, forward primer. | CAT-hph-F | gctcgactcacgtctacccgcgctttcaccgacatggaggcccagaataccctccttg |
| Amplify deletion cassette for *hph* gene from pSH69, reverse primer. | CAT-hph-R | atcattattttacttgaacttttaaccgcatcagtatagcgaccagcattcacatac |
| **Gene validation** |  |  |
| Amplify deletion cassette for *ICL* gene from Po1g-2G-ICLΔ*,* forward primer. | ICL-Hph-knock-F | acgtcagagacaatggtaga |
| Amplify deletion cassette for *ICL* gene from Po1g-2G-ICLΔ*,* reverse primer. | ICL-Hph-knock-R | agcaatcgcgcatatgaaat |
| Amplify deletion cassette for *CAT* gene from Po1g-2G-CATΔ*,* forward primer. | CAT-Hph-knock-F | tgaaatactataattccagc |
| Amplify deletion cassette for *CAT* gene from Po1g-2G-CATΔ*,* reverse primer. | CAT-Hph-knock-R | tgctccatacaagccaacca |

**SUPPLEMENTARY FIGURES**


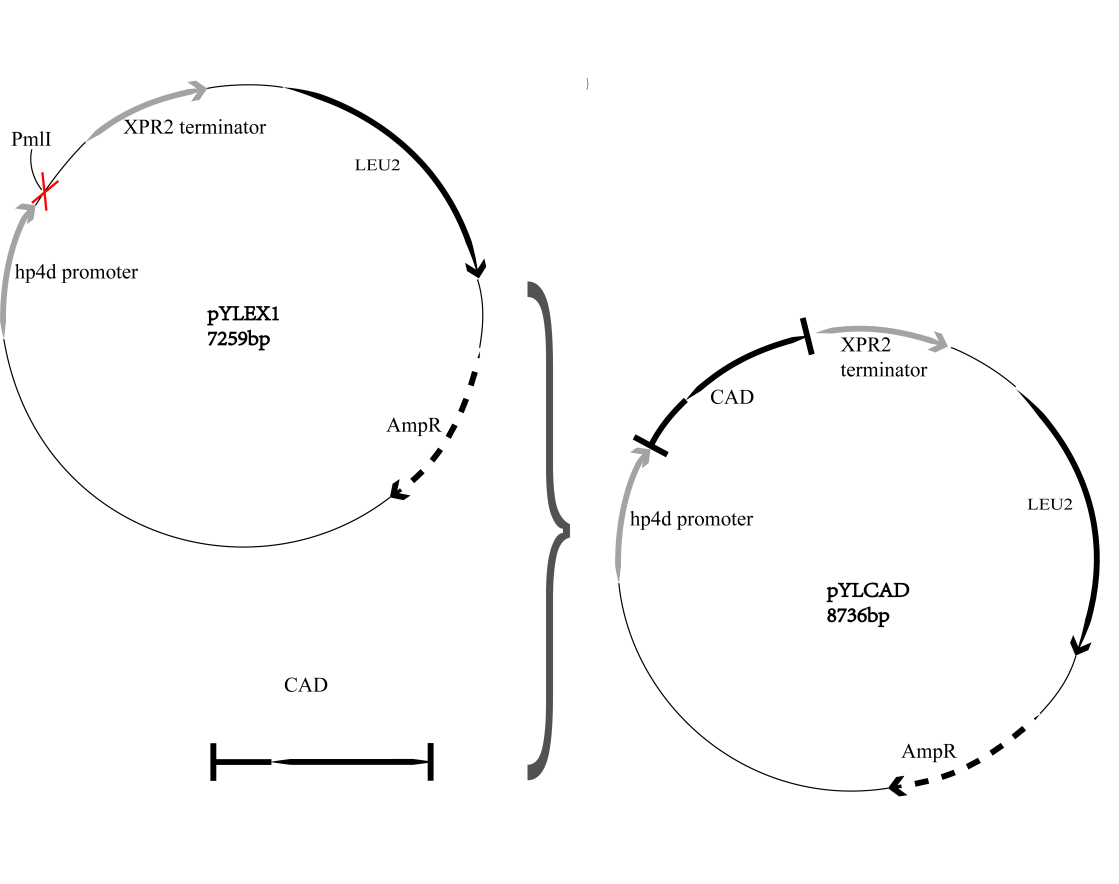


**Figure S1** Assembly of plasmid pYLEX1-CAD. *CAD* gene isolated from *A. terraeus* was cloned into the *Pml I* site of pYLEX1 with a primer pair CAD1-F/CAD2-R to generate plasmid pYLEX1-CAD.


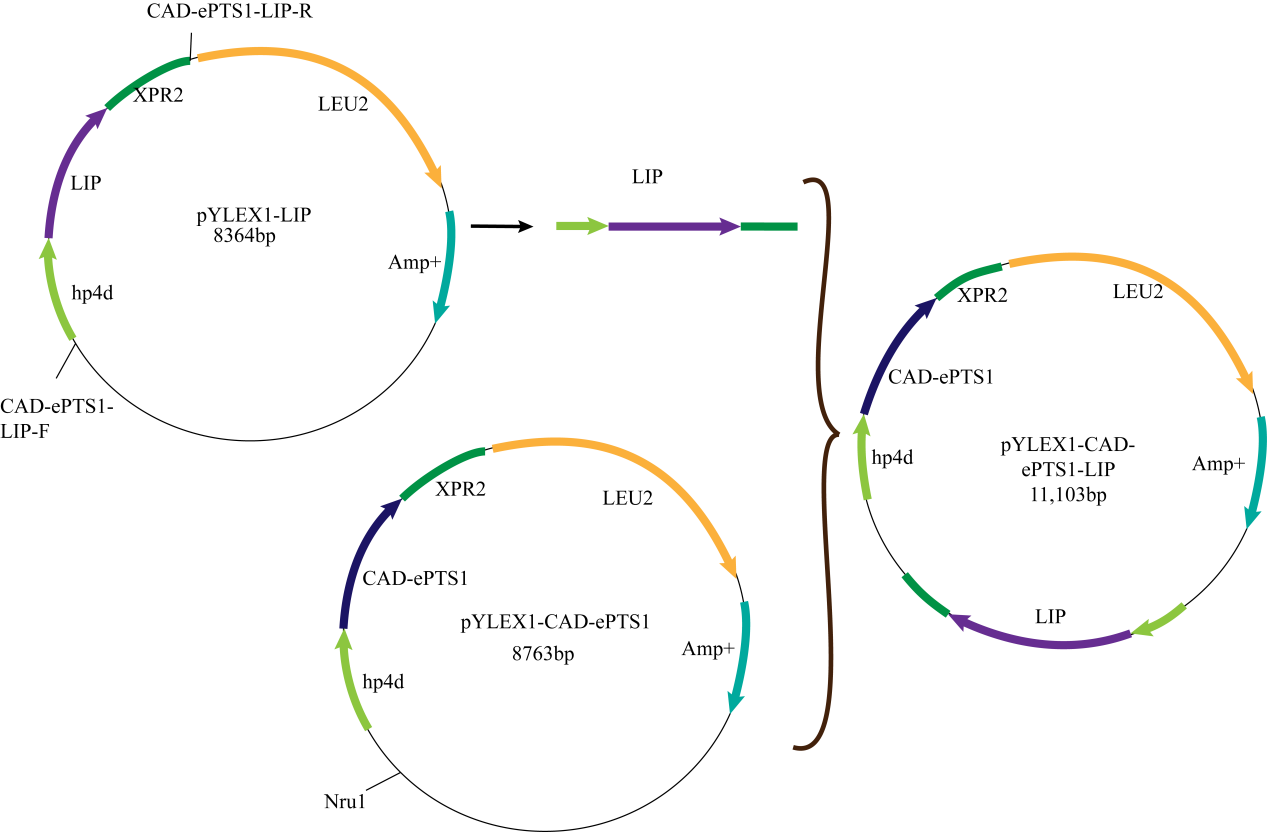


**Figure S2** Assembly of plasmid pYLEX1-CAD-ePTS1-LIP. The expression cassette of *LIP* was cloned into pYLEX1-CAD-ePTS1 with primers CAD-ePTS1-LIP-F/R to generate plasmid pYLEX1-CAD-ePTS1-LIP.

**REFERENCES**

Li, J., Zhu, K., Miao, L., Rong, L. X., Zhao, Y., Li, S. L., et al. (2021). Simultaneous improvement of limonene production and tolerance in *Yarrowia lipolytica* through tolerance engineering and evolutionary engineering. *ACS. Synth. Biol.* 10, 884-896. doi: 10.1021/acssynbio.1c00052
